# Nutritional effect on lipoproteins and their subfractions in patients with Psoriatic Arthritis: a 12-week randomized trial—the DIETA trial

**DOI:** 10.1186/s42358-024-00389-5

**Published:** 2024-06-13

**Authors:** Daniele Scherer, Beatriz Figueiredo Leite, Melissa Aparecida Morimoto, Thauana Luiza Oliveira, Barbara N. Carvalho Klemz, Rosana A. M. Soares Freitas, Caroline Pappiani, Nágila R. Teixeira Damasceno, Marcelo de Medeiros Pinheiro

**Affiliations:** 1 Rheumatology Division, Paulista School of Medicine, Federal University of São Paulo, UNIFESP/EPM), Borges Lagoa Street, 913, room 53, Vila Clementino, São Paulo/SP, 04038-034 Brazil; 2 Nutrition Department, School of Public Health, University of São Paulo, São Paulo, Brazil

**Keywords:** Psoriatic arthritis, Lipoproteins subfractions, HDL cholesterol, LDL cholesterol, Electrophoresis, Nutritional intervention

## Abstract

**Introduction:**

Patients with psoriatic arthritis have some lipid metabolism changes and higher risk of metabolic syndrome (MetS) and cardiovascular diseases, regardless of traditional risk factors, suggesting that chronic inflammation itself plays a central role concerning the atherosclerosis. However, there is a lack of information regarding atherogenic pattern and lipoprotein subfractions burden in these individuals.

**Aim:**

To evaluate the HDL and LDL-cholesterol plasmatic levels and their subfractions after a nutritional intervention in patients with psoriatic arthritis (PsA).

**Methods:**

This was a randomized, placebo-controlled clinical trial of a 12-week nutritional intervention. PsA patients were randomly assigned to 1-Placebo: 1 g of soybean oil daily, no dietetic intervention; 2-Diet + Supplementation: an individualized diet, supplemented with 604 mg of omega-3 fatty acids, three times a day; and 3-Diet + Placebo: individualized diet + 1 g of soybean oil. The LDL subfractions were classified as non-atherogenic (NAth), atherogenic (Ath) or highly atherogenic (HAth), whereas the HDL subfractions were classified as small, medium, or large particles, according to the current recommendation based on lipoproteins electrophoresis.

**Results:**

A total of 91 patients were included in the study. About 62% of patients (*n* = 56) had an Ath or HAth profile and the main risk factors associated were male gender, longer skin disease duration and higher BMI. Thirty-two patients (35%) had a high-risk lipoprotein profile despite having LDL plasmatic levels below 100 mg/dL. The 12-week nutritional intervention did not alter the LDL subfractions. However, there were significant improvement of HDL subfractions.

**Conclusion:**

Recognizing the pro-atherogenic subfractions LDL pattern could be a relevant strategy for identifying PsA patients with higher cardiovascular risk, regardless total LDL plasmatic levels and disease activity. In addition, a short-term nutritional intervention based on supervised and individualized diet added to omega-3 fatty acids changed positively the HDL_LARGE_ subfractions, while LDL_LARGE_ subfraction was improved in hypercholesterolemic individuals.

ClinicalTrials.gov identifier: NCT03142503 (http://www.clinicaltrials.gov/).

## Key messages

• The Ath and HAth profiles are common in patients with PsA, regardless of cholesterol plasmatic levels.

• The lipoprotein subfractions assessment could improve the clinical screening of PsA patients at cardiovascular risk.

• A supervised and individualized nutritional intervention added to omega 3 supplementation may improve the quality of lipoprotein subfractions.

## Introduction

Dyslipidemia is characterized by a wide spectrum of lipid metabolism changes with genetic background and environmental influence [1]. In addition, new information about the classification of lipoproteins (high-density lipoprotein—HDL, intermediate-density lipoprotein—IDL, low-density lipoprotein—LDL, very-low-density lipoprotein—VLDL or chylomicrons) regarding size, density, molecular composition, and functionality, as well as their subfractions have been highlighted to better explain their biological role in health and different diseases [2].

Cardiovascular diseases remain as main cause of mortality worldwide, and many rheumatic diseases are associated with higher cardiovascular mortality, regardless of traditional risk factors, suggesting that chronic inflammation itself plays a central role concerning the atherosclerosis, especially in patients with rheumatic arthritis (RA) [3], systemic lupus erythematous (SLE) [4], psoriatic arthritis (PsA) [5], systemic vasculitis [6] and gout [7].

Patients with PsA have higher prevalence of dyslipidemia, metabolic syndrome (MetS) and cardiovascular mortality than general population [8]. According to the current literature, these patients have low HDL-cholesterol (HDL-c) and high levels of lipoprotein(a) and LDL-cholesterol (LDL-c), mainly related to small subfraction (LDL_3_—the smallest LDL) [9].

Some studies have provided some evidence that small dense LDL particles have the greatest atherogenic potential and higher cardiovascular disease risk, due to easier migration into arterial intima, longer subendothelial retention through link to proteoglycans and higher oxidative susceptibility [10, 11]. Nonetheless, there is a lack of information regarding lipoprotein subfractions burden in patients with rheumatic diseases.

Omega-3 fatty acids may reduce cardiovascular risk [12], including primary and secondary prevention [13, 14], especially related to hypotriglyceridemic effect [15], improvement of lipoproteins quality and anti-inflammatory properties [16]. According to the international guidelines for PsA clinical management and other rheumatic diseases, lifestyle changes have been recommended [17], but there is no a specific nutritional intervention or supplementation defined for these patients. In general, the main dietetic recommendations are based on a healthy diet pattern including low intake of saturated fatty acids and cholesterol, as well as sugar and salt excess avoidance, combined with higher consumption of fibers, whole grains, fruits, and vegetables.

Therefore, our aim was to investigate the relevance of a nutritional counseling with and without omega-3 fatty acid supplementation on plasmatic lipid profile levels and their subfractions in PsA patients through a randomized, controlled, and parallel clinical trial with a 12-week follow-up.

## Methods

### Design study and patients

PsA patients classified according to the Classification Criteria for Psoriatic Arthritis [18] were enrolled in a randomized placebo-controlled clinical trial with a 12-week nutritional intervention [19]. Briefly, the PsA patients were recruited from Sao Paulo’s Hospital (Sao Paulo, Brazil) and other rheumatology facilities (Heliopolis Hospital and Public State Hospital, Sao Paulo, Brazil), according to the eligibility criteria. These details were published previously [19].

Patients with gastrointestinal disorders; dementia; cancer; endocrine, lung, kidney, infectious and neuromuscular diseases were excluded, as well as pregnant and lactating women. Individuals on anabolic steroids or protein, vitamin or antioxidant supplements were not also included. Patients with shellfish or fish previous allergy, or who had changed their pharmacological treatment or physical activity (type, frequency, or modality) in the last six months or had undergone other nutritional therapy in the last 12 months were also excluded [19].

**Fig. 1:**
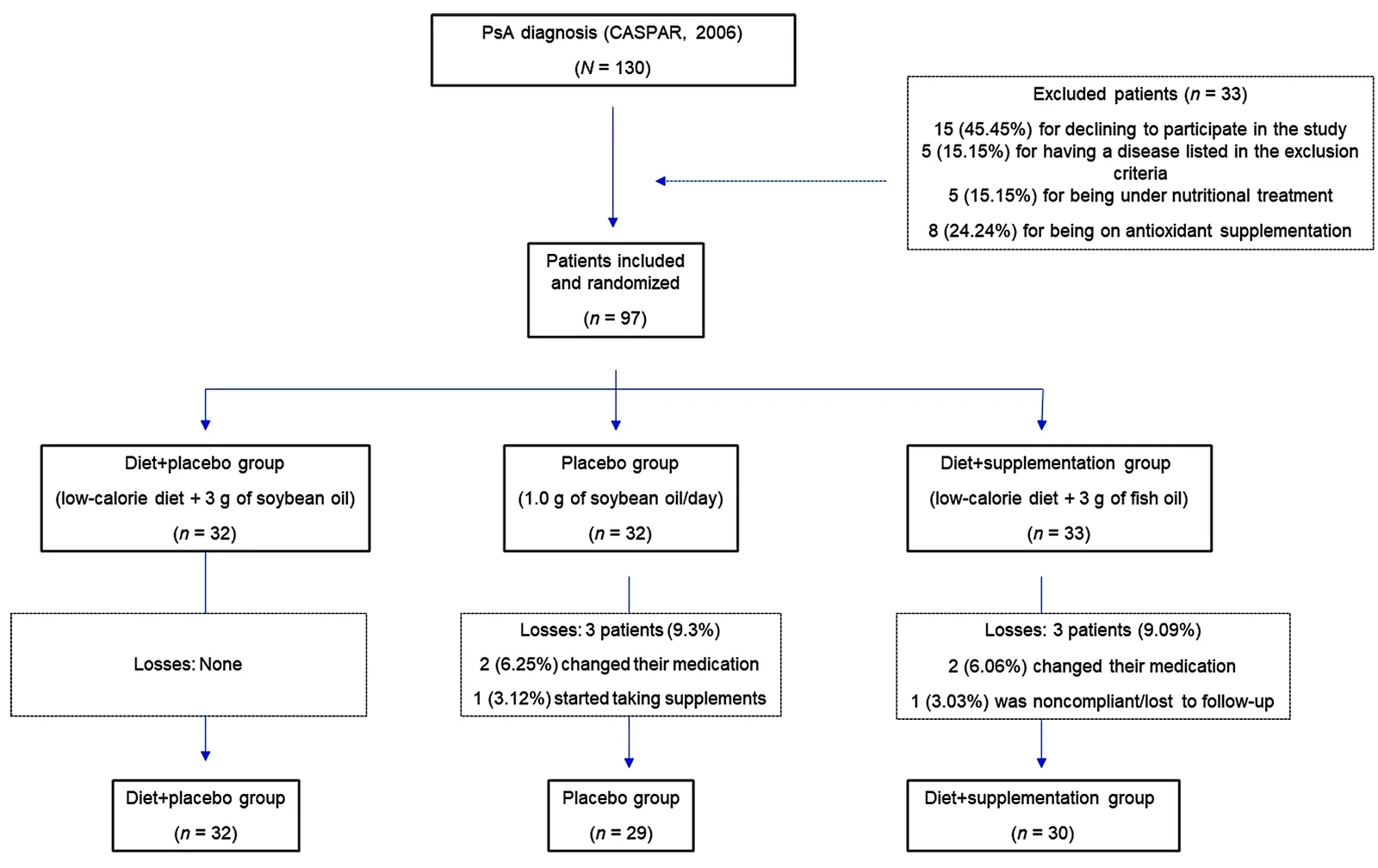
Flow chart of the study population. *CASPAR* Classification Criteria for Psoriatic Arthritis [23]

After eligibility criteria, the patients were included in this randomized clinical trial. Briefly, the patients were randomly divided in three groups according to the nutritional intervention: Placebo (P, *n* = 32)—1 g of soybean oil three times a day and no nutritional intervention; Diet + Supplementation (D + S, *n* = 33)—an individualized and supervised diet plus omega-3 fatty acid supplementation (362 mg of eicosapentaenoic acid—EPA and 242 mg of docosahexaenoic acid—DHA) three times a day, and Diet + Placebo (D + P, *n* = 32)—an individualized and supervised diet (similar to D + S group) with 1 g of soybean oil three times a day. The Fig. 1 shows the flowchart of study and groups under intervention. More information about the dietary plan, including the dietary inflammatory index (DII) and randomization, may be obtained in a previous publication [19].

A total of 96 (at baseline) and 91 (after 12-week intervention) samples from 97 randomized patients were analyzed for HDL and LDL subfractions, respectively. After a 12-week intervention, the dropout rate was 6.2% (*n* = 6). The clinical protocol was approved by Human Ethic Committee of Research at the UNIFESP (CAAE: 00591412.5.0000.5505) and register at Clinical trial (www.clinicaltrials.gov) [NCT03142503]). All procedures were started only after to clarify the study to patient and to collect the consent inform signed.

### Demographic and clinical profile of patients

At baseline and after 12-week intervention, four trained rheumatologists applied a standardized clinical questionnaire, including details about age, gender, smoking status and other lifestyle habits, body mass index (BMI, in kg/m^2^), comorbidities and waist circumference (WC, in cm), as well as information regarding disease activity and current medications.

The 2005 International Diabetes Federation (IDF) classification criteria was used to define metabolic syndrome [20]. The skin and joint outcomes were evaluated by using Psoriasis Area and Severity Index (PASI) [21], Minimal Disease Activity (MDA) [22] and the Disease Activity Score (DAS28) [23].

### Nutritional intervention

The supervised and individualized dietetic intervention was planned according to nutritional status using the Harris-Benedict equation, considering age, sex, weight, and physical activity level [24] as well as eating and cultural habits, food preferences, allergies, socioeconomic status, feasibility, time for preparing the meals and willingness to change the daily routine [19]. For obese and overweight patients, the recommended calories aimed a 5–10%-weight loss with reduction of 500 calories from the estimated total energy expenditure. Although no caloric restriction has been performed for normal-weight patients, the diet plan was adjusted in terms of quality and proportions of macro- and micronutrients for both groups, according to the Dietary Reference Intakes (DRIs) reference values [25].

Patients who were allocated to the Placebo group were guided to maintain their habitual diet. The patients were also instructed not to modify their levels of physical activity and not to change the medications used for the treatment of their underlying diseases, including synthetic conventional and biologic DMARDs (Disease-Modifying AntiRheumatic Drugs).

### Biochemical analysis

At baseline and after the nutritional intervention, venous blood samples (50 ml) were collected in the morning, after a 12 h fast, and frozen at − 80 °C. Triglycerides, total cholesterol and LDL and HDL-cholesterol plasmatic levels were analyzed using standard method and automated system.

The plasmatic levels of VLDL, remnant particles, IDL, LDL and HDL subfractions were analyzed by commercial automated electrophoresis kit (Lipoprint system; Quantimetrix, Redondo Beach, CA, USA). The LDL was separated into seven subfractions, ranging from LDL_1-2_ (LDL_LARGE_) to LDL_3-7_ (LDL_SMALL_). From these results, the mean size was defined in nanometers (nm) and phenotypes A and non-A were calculated after applying the cut-off point (268 nm). The sum of VLDL, remnant particles and IDL was named as atherogenic pattern. HDL was separated into 10 subfractions, ranging from large (HDL_1-3_, HDL_LARGE_) and intermediate (HDL_4-7_, HDL_INTERMEDIATE_) to small (HDL_8-10_, HDL_SMALL_). Results of both LDL and HDL were expressed in percentage of subfractions (%) or adjusted by total and HDL-cholesterol levels (mg/dL), respectively.

### Statistical analysis

Descriptive statistics (mean and standard deviation for continuous variables; absolute and relative frequencies for categorical variables) was used to characterize the patients and all continuous variables were tested for normal distribution by using the Kolmogorov–Smirnov test.

Repeated-measures analysis of variance (ANOVA) was used to evaluate the clinical variables before and after the intervention, according to intention-to-treat analysis. Kruskal–Wallis’s test, Wilcoxon test, Mann–Whitney test and student *t*-test were also used as appropriate. For lipoproteins subfractions, the effect-size of intervention was tested by ANOVA and Bonferroni post-hoc test for continuous variables. For categorical variables, the chi-squared test was used. All statistical analyses were performed with the SPSS Statistics software package, version 22.0 (IBM Corporation, Armonk, NY, USA), and the level of significance was set as p below 0.05.

## Results

A total of 97 PsA patients were randomized in 3 groups during a 12-week follow-up. They had long-standing disease with high comorbidity rate, especially MetS, mild to moderate disease activity (joint and skin manifestations) and most of them were taking synthetic or biologic DMARDs, with no significant differences among them. Regarding total cholesterol, the D + P group had slightly higher plasmatic levels than other groups while triglycerides levels were lower in D + S group. The other lipid parameters, including LDL subfractions, were similar among 3 groups during the randomization (Table 1).

**Table 1:** Baseline clinical parameters in PsA patients, according to the nutritional intervention groups

Variables	Placebo	Diet + Supplementation	Diet + Placebo	*P*
	(*n* = 32)	(*n* = 33)	(*n* = 32)	
Age (years)	53.8 (10.2)	54.8 (13.7)	51.2 (15.1)	0.59
Female, *n* (%)	18 (54.5)	17 (54.8)	18 (54.5)	1.00
BMI (kg/m^2^)	28.9 (4.9)	29.9 (5.3)	30.5 (6.2)	0.47
Waist circumference (cm)	101.8 (12.7)	103.6 (12.8)	104.1 (14.3)	0.77
Disease duration (months)				
Joint disease	157.1 (133.9)	162.8 (140.1)	147.8 (231.4)	0.18
Skin disease	238.9 (162.8)	216.8 (153.9)	237.4 (227.1)	0.87
Comorbidities, *n* (%)				
Diabetes	8 (24.2)	9 (28.1)	3 (9.4)	0.14
Hypertension	15 (45.5)	15 (46.9)	15 (46.9)	1.00
Dyslipidemia	18 (54.5)	12 (37.5)	13 (39.4)	0.27
MetS, *n* (%)	22 (66.7)	18 (58.1)	13 (39.4)	0.07
Concomitant medications related to MetS				
Insulin	2 (6.1)	4 (12.5)	1 (3.1)	0.33
Statins	10 (30.3)	17 (53.1)	6 (18.7)	**0.01**
Metformin	7 (21.2)	10 (31.2)	3 (9.4)	0.09
Antihypertensive	14 (42.4)	16 (50.0)	16 (50.0)	0.84
MET-minutes/week	945.1 ± 2544.7	781.3 ± 1366.9	817.9 ± 2167.3	0.95
Dietary Inflammatory Index	2.4 (0.9)	2.2 (1.0)	2.8 (0.8)	0.50
PASI	2.51 (3.79)	3.41 (6.10)	3.49 (6.31)	0.92
DAS28-CRP	2.93 (1.19)	2.83 (1.55)	2.98 (1.35)	0.75
Glucocorticoid use, *n* (%)	5 (15.2)	3 (9.4)	3 (9.4)	0.66
NSAID use, *n* (%)	5 (15.2)	5 (15.6)	2 (6.3)	0.42
Synthetic DMARDs, *n* (%)	18 (54.5)	23 (71.8)	23 (71.8)	0.23
TNF inhibitors, *n* (%)	7 (21.2)	8 (25)	6 (18.7)	0.83
Lipid profile (mg/dL)				
Total cholesterol	196.6 (37.2)	184.9 (44.5)	205.9 (44.2)^a^	**0.05**
LDL-c	115.5 (27.2)	115.3 (41.4)	130.6 (38.5)	0.08
HDL-c	45.1 (12.0)	48.7 (11.1)	49.5 (10.8)	0.75
Triglycerides	166.2 (105.8)	112.11 (40.7)^a^	137.7 (91.1)	**0.03**

*PASI* Psoriasis Area Severity Index, *DAS28-CRP* 28-joint DAS based on C-reactive protein levels, *NSAID* Non-steroidal anti-inflammatory drug, *DMARDs* Disease-modifying anti-rheumatic drugs, *LDL-c* Low-density lipoprotein cholesterol, *HDL-c* High-density lipoprotein cholesterol, *MetS* Metabolic syndrome, *METs* Metabolic Equivalents using the IPAQ (International Physical Activity Questionnaire)

^a^Significant differences to the other groups

Considering the LDL subfractions, women showed a tendency to have higher LDL_LARGE_ levels than men (43.7 mg/dL *versus* 38.0 mg/dL; *p* = 0.063). Opposite profile was observed for LDL_SMALL_, in which men had higher mean LDL_SMALL_ levels than women (13.6 mg/dL *versus* 8 mg/dL; *p* = 0.015). The Atherogenic pattern was more frequent in overweight and obese patients than in those with BMI within normal range (< 25 kg/m^2^); *p* = 0.048). The other traditional risk factors for cardiovascular events and clinical data, including disease activity endpoints and medications did not have significant differences among the groups. Patients on glucocorticoids had lower LDL_SMALL_ (7.2 mg/dL *versus* 11.0 mg/dL, *p* = 0.046), and, consequently, lower frequency of phenotype A. Patients aged more than 50 years also had higher levels of LDL_LARGE_ than younger ones (53.7 mg/dL *versus* 45.8 mg/dL, *p* = 0.03). On the other hand, the atherogenic pattern and LDL_SMALL_ were significantly less common among the patients who had skin disease for ≤ 10 years. In addition, all PsA patients had higher values of atherogenic subfractions (LDL_SMALL_ = 5.4%; HDL_SMALL_ = 35.7%; Atherogenic pattern = 19%), regardless of total cholesterol plasmatic levels. Of the 91 patients tested for LDL subfractions, 56 (62%) had Atherogenic pattern or LDL_SMALL_. Interestingly, only 32 patients (35%) that were classified as having this profile had LDL-c levels ≥ 130 mg/dL. In contrast, less than 10% (*n* = 6) of patients with LDL-c levels below 130 mg/dL had a normal lipoprotein pattern (Table 2).

**Table 2:** Association among clinical variables and small and large LDL subfractions and Atherogenic pattern in PsA patients at baseline

Variables	Atherogenic pattern (m/SD)		Small LDL (m/SD)		Large LDL (m/SD)		Phenotype non-A (< 268 nm)	Phenotype A (≥ 268 nm)
	(%)	(mg/dL)	(%)	(mg/dL)	(%)	(mg/dL)	(*n*, %)	(*n*, %)
Age								
≤ 50 years (*n* = 36)	18.9 (6.6)	36.3 (15.8)	6.1 (5.0)	11.9 (10.7)	20.1 (5.1)	37.2 (10.4)	26 (28.6)	10 (11.0)
> 50 years (*n* = 55)	19.1 (9.4)	38.8 (27.2)	4.9 (5.2)	9.7 (10.8)	21.9 (5.0)	43.6 (16.3)	27 (29.7)	28 (30.8)
*p-value*	0.71^b^	0.86^b^	0.174^b^	0.226^b^	0.089^a^	**0.024** ^**a**^		**0.029** ^**d**^
Sex								
Male (*n* = 42)	20 (11.0)	39.9 (31.1)	6.9 (5.7)	13.6 (12.2)	20.3 (5.7)	38.0 (15.0)	30 (33.0)	12 (13.2)
Female (*n* = 49)	18 (5.0)	36 (13.5)	4.0 (4.2)	8.0 (8.6)	22.0 (4.4)	43.7 (13.6)	23 (25.3)	26 (28.6)
*p-value*	0.71^b^	0.96^b^	**0.006** ^**b**^	**0.015** ^**b**^	0.117^a^	0.063^a^		**0.018** ^**d**^
Joint disease time								
≤ 5 years (*n* = 31)	19.7 (6.6)	36.4 (14.1)	5.7 (4.6)	10.6 (10.0)	21.1 (4.0)	38.5 (9.6)	22 (24.2)	9 (9.9)
> 5 years (*n* = 60)	18.7 (9.2)	38.6 (26.9)	5.2 (5.4)	10.6 (11.2)	21.3 (5.6)	42.4 (16.4)	31 (34.1)	29 (31.9)
*p-value*	0.36^b^	0.99^b^	0.295^b^	0.635^b^	0.865^a^	0.160^a^		0.077^d^
Skin disease time								
≤ 10 years (*n* = 30)	20.2 (6.0)	38.8 (15.5)	7.1 (6.0)	13.5 (12.3)	20.9 (5.2)	39.4 (13.2)	21 (23.1)	9 (9.9)
> 10 years (*n* = 61)	18.4 (9.3)	37.3 (26.3)	4.5 (4.5)	9.1 (9.7)	21.3 (5.1)	42.0 (15.1)	32 (35.2)	29 (31.9)
*p-value*	**0.03** ^b^	0.28^b^	**0.038** ^**b**^	0.099^b^	0.714^a^	0.427^a^		0.111^d^
Smoking								
No (*n* = 77)	19.1 (8.8)	37.9 (24.7)	5.4 (5.0)	10.6 (10.5)	21.5 (5.3)	41.8 (15.4)	45 (50.0)	32 (35.6)
Yes (*n* = 13)	18.9 (6.0)	36.9 (13.7)	5.6 (6.1)	11.0 (12.7)	19.4 (3.7)	36.9 (7.7)	8 (8.9)	5 (5.6)
*p-value*	0.8^b^	0.73^b^	0.809^b^	0.601^b^	0.161^a^	0.089^a^		0.834^d^
MetS								
No (*n* = 67)	19.4 (9.2)	38 (26.0)	5.5 (5.2)	10.5 (10.8)	20.9 (5.2)	39.1 (12.3)	40 (44.0)	27 (29.7)
Yes (*n* = 24)	17.8 (5.2)	37.1 (13.1)	5.0 (4.9)	10.7 (10.7)	22.1 (4.7)	46.6 (18.6)	13 (14.3)	11 (12.1)
*p-value*	0.59^b^	0.54^b^	0.935^b^	0.787^b^	0.322^a^	0.078^a^		0.637^d^
BMI								
≤ 25 kg/m^2^ (*n* = 14)	16.4 (5.6)	32.2 (14.9)	5.4 (5.6)	10.7 (11.6)	22.6 (5.1)	42.9 (16.3)	8 (8.9)	6 (6.7)
> 25 kg/m^2^(*n* = 76)	19.5 (8.8)	39 (24.5)	5.4 (5.1)	10.7 (10.7)	20.9 (5.1)	40.7 (14.3)	45 (50.0)	31 (34.4)
*p-value*	**0.048** ^b^	0.16^b^	0.938^b^	0.845^b^	0.252^a^	0.611^a^		0.885^d^
PASI								
0 (*n* = 28)	21 (5.6)	36.2 (14.9)	5.9 (6.0)	11.3 (12.1)	21.4 (5.7)	39.9 (12.1)	15 (16.7)	13 (14.4)
≤ 5 (*n* = 47)	20 (10.6)	39.1 (29.5)	5.3 (4.8)	10.4 (10.5)	21.3 (5.0)	40.5 (15.1)	28 (31.1)	19 (21.1)
> 5 (*n* = 15)	16.6 (3.0)	36.5 (13.3)	4.7 (4.8)	10.1 (9.8)	20.6 (4.6)	44.9 (17.1)	10 (11.1)	5 (5.6)
*p-value*	0.71^c^	0.89 ^c^	0.749^c^	0.921^c^	0.882^c^	0.539^c^		0.701^d^
DAS28-CRP								
REM (*n* = 24)	20.3 (13.6)	37.8 (39.5)	6.1 (5.1)	11.0 (11.2)	22.4 (5.7)	38.2 (14.5)	17 (24.6)	7 (10.1)
LAT (*n* = 15)	18.3 (4.6)	39.2 (15.3)	4.6 (4.6)	10.0 (9.9)	21.9 (4.2)	46.4 (15.9)	7 (10.1)	8 (11.6)
MHAT (*n* = 29)	18.7 (5.7)	36.4 (11.8)	5.1 (5.1)	9.9 (10.3)	21.0 (4.7)	41.3 (13.4)	17 (24.6)	13 (18.8)
*p-value*	0.74 ^c^	0.16 ^c^	0.639^c^	0.920^c^	0.591^c^	0.228^c^		0.301^d^
DMARDs								
No (*n* = 29)	18.9 (6.5)	36.2 (15.6)	5.8 (4.8)	11.0 (9.9)	21.5 (5.9)	40.5 (13.5)	17 (18.9)	12 (13.3)
Yes (*n* = 61)	19.1 (9.2)	38.6 (26.4)	5.2 (5.3)	10.5 (11.3)	21.1 (4.7)	41.3 (15.2)	36 (40.0)	25 (27.8)
*p-value*	0.91^b^	0.83^b^	0.484^b^	0.598^b^	0.748^a^	0.807^a^		0.972^d^
NSAIDs								
No (*n* = 81)	19.2 (8.6)	38.3 (24.2)	5.7 (5.3)	11.2 (11.1)	21.1 (5.2)	41.0 (14.2)	50 (55.6)	31 (34.4)
Yes (*n* = 9)	18 (5.9)	32.8 (13.5)	3.1 (3.2)	5.8 (6.1)	22.4 (4.4)	41.7 (18.9)	3 (3.3)	6 (6.7)
*p-value*	0.66^b^	0.38^b^	0.190^b^	0.151^b^	0.485^a^	0.897^a^		0.100^d^
Statins								
No (*n* = 59)	19.2 (9.5)	38.5 (26.9)	5.4 (5.4)	10.8 (11.5)	21.1 (5.2)	40.8 (14.2)	34 (37.4)	25 (27.5)
Yes (*n* = 32)	18.7 (5.7)	36.5 (14.5)	5.3 (4.7)	10.2 (9.4)	21.4 (4.9)	41.7 (15.3)	19 (20.9)	13 (14.3)
*p-value*	0.57^b^	0.87^b^	0.819^b^	0.806^b^	0.762^a^	0.766^a^		0.872^d^
Glucocorticoids								
No (*n* = 81)	19.4 (8.7)	38.8 (24.2)	5.6 (4.9)	11.0 (10.4)	21.0 (5.2)	41.1 (14.6)	52 (57.8)	29 (32.2)
Yes (*n* = 9)	15.8 (2.8)	28.6 (10.4)	3.9 (7.1)	7.2 (13.9)	22.9 (4.4)	40.6 (14.9)	1 (1.1)	8 (8.9)
*p-value*	0.22^b^	0.24^b^	0.090^b^	**0.046** ^**b**^	0.307^a^	0.912^a^		**0.003** ^**e**^
Biologic DMARDs								
No (*n* = 56)	18.5 (9.2)	36.7 (26.9)	5.2 (5.5)	10.4 (11.8)	21.0 (5.1)	40.3 (14.7)	30 (33.3)	26 (28.9)
Yes (*n* = 34)	20 (7.0)	39.6 (16.1)	5.7 (4.6)	11.0 (9.1)	21.5 (5.1)	42.3 (14.5)	23 (25.6)	11 (12.2)
*p-value*	0.18^b^	0.15^b^	0.343^b^	0.335^b^	0.684^a^	0.546^a^		0.188^d^

LDL_LARGE_ (LDL_1 − 2_); LDL_SMALL_(LDL_3 − 7_)

*MetS* Metabolic syndrome, *PASI* Psoriasis Area Severity Index, *DAS28-CRP* 28-joint DAS based on C-reactive protein levels, *REM* Remission, *LDA* Low disease activity, *MHAT* Moderate to high activity, *DMARDs* Disease-modifying antirheumatic drugs, *NSAID* Non-steroidal anti-inflammatory drug

*Atherogenic pattern (sum of VLDL, remnants and IDL fractions)

^a^Student’s *t*-test

^b^Mann–Whitney test

^c^Kruskal–Wallis test

^d^One-way ANOVA

Regarding HDL subfractions, male patients and those with MetS had higher content of HDL_SMALL_ particles. Nevertheless, the most protective subfractions (HDL_INTERMEDIATE_ and HDL_LARGE_ particles) were significantly more common in women, as well as in those who had low disease activity or were in remission and were not being treated with biologic agents (15.1 mg/dL *versus* 12.5 mg/dL, *p* = 0.03), even after statistical adjustment for statins. Most of the patients had adequate HDL values (≤ 40 mg/dL), however, the HDL_LARGE_ was found in only 10% of sample, especially in patients with low disease activity (Table 3).

**Table 3:** Association among clinical variables and the HDL subfractions pattern, according to the particle size, in PsA patients at baseline

	HDL_SMALL_		HDL_IMTERMEDIATE_		HDL_LARGE_	
	(%)	(mg/dL)	(%)	(mg/dL)	(%)	(mg/dL)
Age						
≤ 50 years (*n* = 40)	27.6 (9.0)	12.9 (5.3)	43.6 (5.2)	20.4 (5.8)	28.8 (10.1)	13.2 (5.5)
> 50 years (*n* = 56)	24.4 (8.0)	11.9 (5.2)	45 (5.4)	21.9 (5.8)	30.6 (9.1)	14.9 (6.1)
*p*	0.07^a^	0.38^b^	0.21^a^	**0.04** ^b^	0.37^a^	0.16^a^
Gender						
Male (*n* = 43)	25.2 (6.2)	11.1 (4.4)	45.1 (5.5)	20 (5.8)	29.7 (8.1)	12.9 (4.8)
Female (*n* = 53)	26.1 (10.1)	13.2 (5.7)	43.9 (5.2)	22.3 (5.7)	30 (10.5)	15.2 (6.5)
*p*	0.6^a^	**0.04** ^b^	0.28^a^	**0.048** ^a^	0.9^a^	0.05^a^
Joint disease duration						
≤ 5 years (*n* = 34)	25.8 (8.6)	11.9 (4.7)	44.4 (4.7)	20.6 (5.1)	29.8 (8.8)	14 (6.0)
> 5 years (*n* = 63)	25.7 (8.6)	12.5 (5.6)	44.5 (5.7)	21.6 (6.2)	29.8 (9.9)	14.3 (5.8)
*p*	0.97^a^	0.78^b^	0.95^a^	0.45^a^	0.1^a^	0.84^a^
Skin disease duration						
≤ 10 years (*n* = 34)	26.8 (9.6)	12.5 (5.1)	44.5 (5.3)	20.8 (4.8)	28.7 (8.4)	13.6 (5.8)
> 10 years (*n* = 62)	25.1 (7.9)	12.1 (5.4)	44.4 (5.4)	21.5 (6.4)	30.5 (10.0)	14.5 (5.9)
*p*	0.36^a^	0.74^b^	0.89^a^	0.57^a^	0.37^a^	0.45^a^
Current Smoking						
No (*n* = 82)	25.8 (8.9)	12.3 (5.2)	44.1 (5.3)	21.1 (5.6)	30 (9.6)	14.3 (6.0)
Yes (*n* = 13)	24.9 (6.2)	12.2 (6.1)	46.5 (5.8)	22.2 (7.8)	28.6 (9.7)	13.2 (5.6)
*p*	0.73^b^	0.74^b^	0.14^a^	0.51^a^	0.61^a^	0.55^a^
MetS						
No (*n* = 71)	24.3 (7.2)	11.2 (3.8)	44.5 (5.4)	20.6 (5.0)	31.1 (7.9)	14.4 (5.1)
Yes (*n* = 25)	29.6 (10.7)	15.5 (7.3)	44.1 (5.4)	23.1 (7.6)	26.2 (12.4)	13.6 (7.7)
*p*	**0.03** ^b^	**0.01** ^b^	0.36^b^	0.41^b^	0.07^a^	0.55^a^
BMI						
≤ 25 kg/m^2^(*n* = 15)	25.9 (6.5)	13.7 (5.8)	45 (5.6)	23.6 (3.4)	29.2 (9.6)	15.1 (6.2)
> 25 kg/m^2^(*n* = 80)	25.6 (8.9)	12 (5.2)	44.3 (5.3)	20.8 (5.6)	30.1 (9.5)	14.1 (5.8)
*p*	0.93^a^	0.23^b^	0.64^a^	0.09^a^	0.42^a^	0.51^a^
PASI						
0 (*n* = 30)	24.9 (8.7)	11.4 (4.3)	44.9 (6.6)	20.7 (4.5)	30.2 (8.2)	14.2 (5.7)
≤ 5 (*n* = 48)	25.8 (8.4)	10.8 (5.7)	43.9 (5.0)	21.3 (7.1)	30.3 (10.2)	14.4 (6.2)
> 5 (*n* = 17)	26.9 (9.2)	13.2 (5.8)	45.2 (4.1)	21.8 (4.3)	28 (10.0)	13.4 (5.7)
*p*	0.74^c^	0.48^c^	0.61^c^	0.82^c^	0.68^c^	0.83^c^
DAS28-CRP						
REM (*n* = 25)	26 (8.7)	10.9 (3.7)	43.9 (6.6)	19 (4.7)	30 (8.7)	13.1 (5.4)
LDA (*n* = 15)	23.1 (4.8)	12 (4.1)	43.3 (4.3)	22.1 (4.7)	33.7 (7.5)	17.6 (6.1)
MHAT (*n* = 55)	25.9 (8.6)	12.4 (5.8)	44.1 (4.1)	20.8 (6.3)	30 (9.7)	13.7 (5.5)
*p*	0.46^c^	0.51^c^	0.86^c^	0.2^c^	0.37^c^	**0.04** ^c^
DMARDs						
No (*n* = 32)	24.9 (8.9)	11.6 (5)	43.4 (5.2)	20.4 (5.4)	31.8 (10)	14.7 (5.9)
Yes (*n* = 63)	26.1 (8.5)	12.6 (5.5)	45 (5.4)	21.7 (6.1)	28.9 (9.2)	13.9 (5.9)
*p*	0.31^b^	0.35^a^	0.15^a^	0.41^b^	0.16^a^	0.52^b^
NSAIDs						
No (*n* = 84)	26 (8.9)	12.5 (5.5)	44.5 (5.7)	21.4 (6.0)	29.4 (9.9)	14 (6.1)
Yes (*n* = 11)	23.3 (4.4)	10.3 (2.1)	44 (3.3)	20 (4.3)	32.8 (5.6)	15.3 (5.5)
*p*	0.29^b^	0.14^b^	0.54^a^	0.51^b^	0.27^a^	0.5^a^
Statins						
No (*n* = 64)	26.1 (8.9)	12.8 (6.0)	44.6 (4.9)	21.7 (6.4)	29.3 (10.3)	14 (5.9)
Yes (*n* = 32)	25 (7.8)	11.3 (3.4)	44.2 (6.2)	20.3 (4.6)	30.8 (7.6)	14.5 (5.8)
*p*	0.59^b^	0.38^b^	0.72^a^	0.26^a^	0.47^a^	0.72^a^
Glucocorticoids						
No (*n* = 84)	25.8 (8.9)	12.2 (5.4)	44.2 (5.5)	21 (5.9)	29.9 (9.8)	14 (6.0)
Yes (*n* = 11)	24.5 (5.6)	12.5 (4.7)	46.3 (3.8)	23.4 (5.8)	29.2 (7.2)	15.1 (5.3)
*p*	0.78^b^	0.51^b^	0.11^b^	0.2^a^	0.81^a^	0.57^a^
Biologic DMARD						
No (*n* = 59)	25.6 (8.5)	12.8 (5.5)	44 (4.9)	22.2 (6.5)	30.4 (9.5)	15.1 (6.1)
Yes (*n* = 36)	25.9 (8.9)	11.4 (4.9)	45.2 (6.1)	19.7 (4.3)	28.9 (9.7)	12.5 (5.1)
*p*	0.83^b^	0.2^b^	0.33^a^	**0.04** ^a^	0.48^a^	**0.03** ^a^

*HDL* High-density lipoprotein, *MetS* Metabolic syndrome, *PASI* Psoriasis Area Severity Index, *DAS28-CRP* 28-joint DAS based on C-reactive protein levels, *DMARDs* Disease-modifying antirheumatic drugs, *NSAID* Non-steroidal anti-inflammatory drugs, *REM* Remission, *LDA* Low disease activity, *MHAT* Moderate to high activity

^a^Student’s *t*-test

^b^Mann–Whitney test

^c^One-way ANOVA

**Fig. 2:**
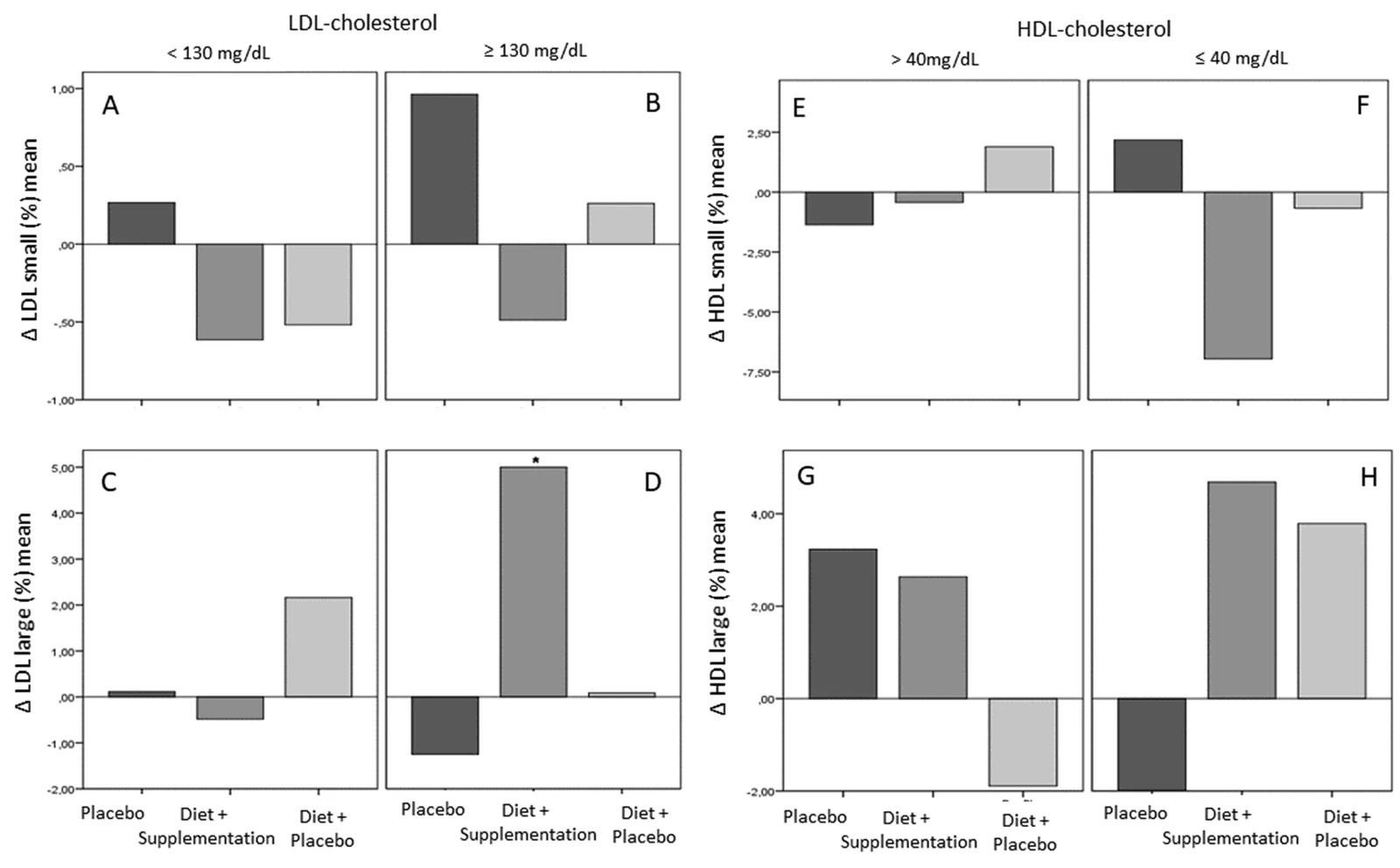
HDL- and HDL-cholesterol plasmatic levels changes according to the nutritional intervention groups and cut-off points for LDL-c and HDL-c in PsA patients. ^*^Differences inter group tested one-way ANOVA and post hoc Tukey

**Fig. 3:**
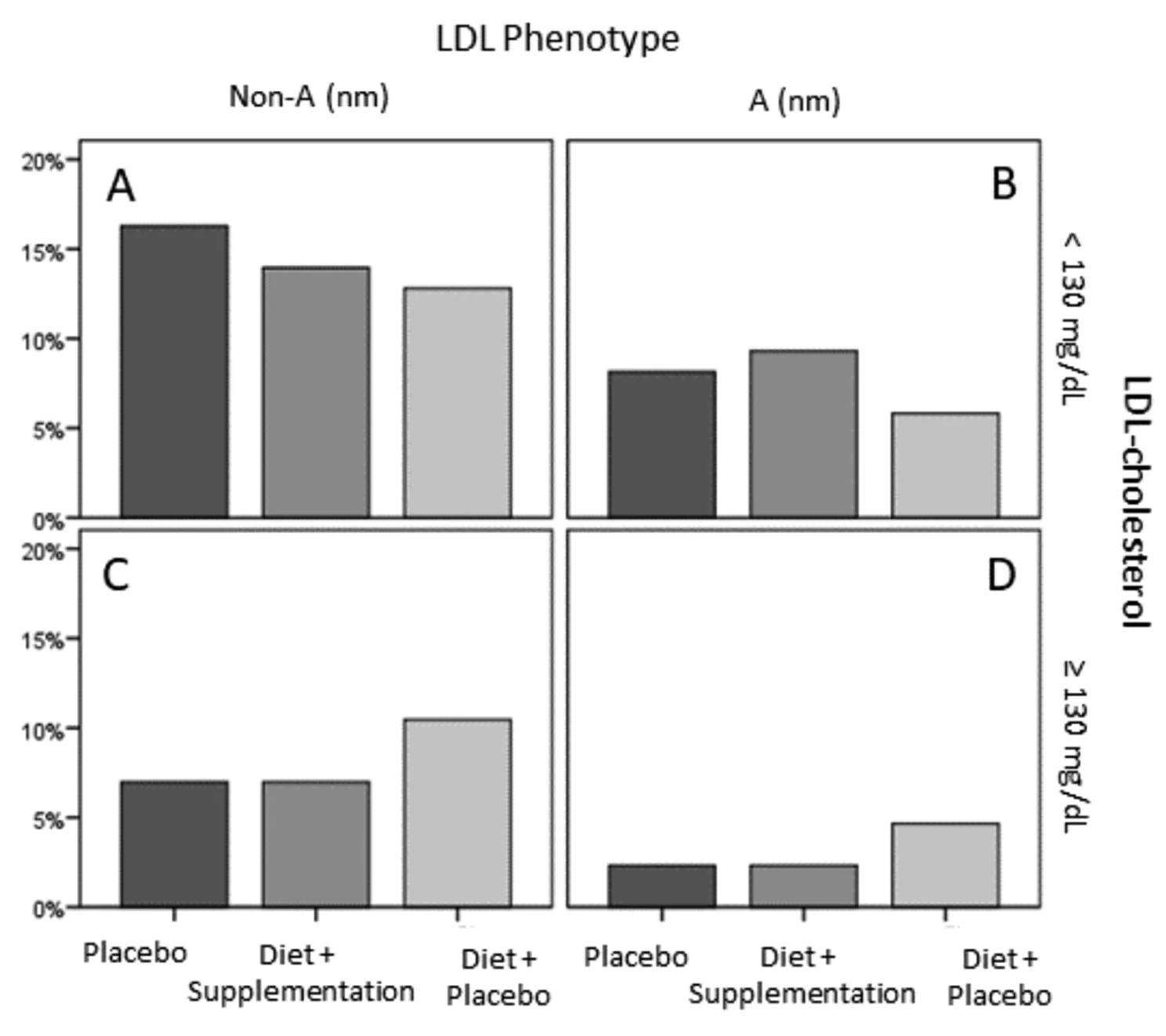
LDL-phenotype, according to the nutritional intervention and cut-off points for LDL-cholesterol in PsA patients. ^*^Differences inter group tested one-way ANOVA and post hoc Tukey. Significant level adopted: *p* < 0.05

After a 12-week nutritional intervention, the D + S group had significant higher increment of LDL_LARGE_ in patients with LDL-c plasmatic values ≥ 130 mg/dL at baseline (Fig. 2A–D). Patients from the D + S (*p* = 0.059) and D + P (*p* = 0.062) groups showed trend to increase HDL_LARGE_ in patients with low HDL-c (< 40 mg/dL) when compared to Placebo group (Fig. 2E–H). Also, HDL_LARGE_ increased in all groups after intervention. Although an expected reduction in Atherogenic pattern was observed in Placebo group, this group showed 118% increases in HDL_SMALL_ compared to baseline, while other groups not changed after intervention (Table 4). On the other hand, there were not any significant changes regarding the phenotypes non-A and A, even after LDL-c classification by cut-off of 130 mg/ dL (Fig. 3A–D). After nutritional intervention, there was a significant reduction of DII in 3 groups [placebo: −1.4 (1.1); D + S: −1.1 (1.0); D + P: −1.8 (1.3)] concomitantly to improvement quality of lipoprotein subfractions.

**Table 4:** Distribution of small and large LDL subfractions, according to the nutritional intervention groups

Variables	At baseline	After 12 weeks	*p*-value^b^
LDL_SMALL_ (mg/dL)			
All	10.6 (5.7)		
All (%)	5.4		
Placebo	13.2 (13.0)	13.7 (12.5)	0.801
Diet + Supplementation	8.4 (9.5)	7.5 (7.8)	0.600
Diet + Placebo	10.1 (9.1)	10.8 (9.0)	0.635
*p-value*^*a*^	0.211	0.060	
LDL_LARGE_ (mg/dL)			
All	50.6 (18.8)		
All (%)	26.0		
Placebo	40.3 (14.5)	39.7 (12.2)	0.783
Diet + Supplementation	41.3 (14.3)	41.9 (18.3)	0.818
Diet + Placebo	41.7 (15.1)	42.0 (10.6)	0.917
*p-value*^*a*^	0.936	0.780	
HDL_LARGE_ (mg/dL)			
All	14.2 (5.9)		
All (%)	29.8		
Placebo	13.2 (5.5)	29.3 (13.1)	**0.030**
Diet + Supplement	14.6 (6.4)	32.3 (9.4)	**0.035**
Diet + Placebo	14.7 (5.8)	29.1 (10.3)	**0.019**
*p-value*^*a*^	0.538	0.448	
HDL_INTERMEDIATE_ (mg/dL)			
All	21.3 (5.3)		
All (%)	44.4		
Placebo	20.4 (6.6)	40.7 (10.5)	0.860
Diet + Supplement	21.6 (5.0)	42.8 (5.0)	0.314
Diet + Placebo	21.8 (5.8)	42.9 (6.4)	0.256
*p-value*^*a*^	0.555	0.430	
HDL_SMALL_ (mg/dL)			
All	12.3 (5.3)		
All (%)	35.7		
Placebo	11.8 (6.2)	24.4 (12.7)	0.029
Diet + Supplement	12.7 (5.2)	24.1 (7.5)	0.560
Diet + Placebo	12.4 (4.5)	26.1 (9.4)	0.720
*p-value*^*a*^	0.785	0.700	
Atherogenic pattern (mg/dL)*			
All	37.8 (23.3)		
All (%)	19.0		
Placebo	43.3 (34.7)	40.4 (16.6)	0.007
Diet + Supplement	34.6 (15.3)	32.9 (12.4)	0.001
Diet + Placebo	35.6 (13.7)	33.3 (11.3)	0.244
*p-value*^*a*^	0.285	0.060	

Significant level adopted: *p* < 0.05

^a^Differences inter group tested one-way ANOVA and post hoc Turkey

^b^Differences intra group performed by Student’s *t* test

*Atherogenic pattern (sum of VLDL, remnants and IDL fractions)

## Discussion

Our results showed a high prevalence of atherogenic lipoprotein subfractions in PsA patients, regardless of dyslipidemia level, suggesting that monitoring them could be a promising strategy to improve the cardiovascular screening and stratification for patients at risk. Furthermore, dietetic counseling and omega-3 supplementation promoted significant atherogenic subfractions changes in short-term, highlighting the increment in HDL_LARGE_ particles level, while patients under placebo had increased HDL_SMALL_.

Nutritional counseling is part of a global strategy for prevention and treatment of some rheumatic diseases [26] what was addressed and reinforced in our clinical trial. Although the pivotal study recently published [19] have not demonstrated that omega 3 supplementation provided any significant improvement regarding skin or joint disease activity, it was able to promote some qualitative lipoproteins changes in PsA patients, suggesting some anti-atherogenic properties of fatty acids [15]. Considering that the reduced atherogenic pattern is composed basically by triglycerides-rich lipoproteins (VLDL, remnants and IDL fractions), the omega-3 supplementation may modulate triglycerides levels by stimulus of expression and synthesis of lipoprotein lipase [27]. However, in inflammatory conditions, as occur in PsA patients, the cytokines induce insulin resistance and consequent activation of hormone-sensitive lipase and hypertriglyceridemia [28] as observed in 72% of PsA patients at baseline (cut-off point 150 mg/dL). Conversely, individuals from placebo group also showed atherogenic pattern reduction, that can be, at least in part, explained by potential positive feedback for being participating of a clinical trial and some dietetic modification cannot be completely excluded, especially by fortnightly appointments with the medical team. Also, it is important to state that during a 12-week follow-up, no non-pharmacological intervention (diet and physical activity, smoking, and alcohol intake) or medications changes were allowed.

Hypertriglyceridemia is the most potent stimulus to cholesterol ester transport protein (CETP) activity and, in acute inflammation animal models, the CETP activity was even more increased [29]. CETP modulates the exchange of triglycerides from lipoprotein-rich in apolipoprotein B (Apo B) to HDL particles, reducing its main cardioprotective function—the reverse cholesterol transport [30]. In our study, the mean of triglycerides in both, Diet + Supplementation and Diet + Placebo groups, were below the cut-off point for adequate level (150 mg/dL), therefore, the response to diet and omega-3 interventions cannot be explained by triglycerides levels at baseline. Induced-diet modifications, regardless of omega-3 fatty acids, are in line with improvement in reverse cholesterol transport hallmarked by increased HDL_LARGE_. In fact, placebo group significant changes in HDL_LARGE_ subfractions, but the increasing in HDL_SMALL_ (118%), certainly compromises the HDL functionality. In addition to modulation of reverse cholesterol transport, recently we observed that omega-3 fatty acids intervention modified HDL size trough changes in fatty acid composition and non-esterified fatty acids (NEFAS) content in this lipoprotein. Both events contributed to increase in HDL_LARGE_ after 8-weeks of intervention (3 g/day; 60% eicosapentaenoic—EPA and docosahexaenoic—DHA) [31]—a similar quantity of omega-3 fatty acids used for PsA patients.

Regarding the high prevalence of dyslipidemia associated to high total cholesterol and triglycerides at baseline, we evaluated the response to nutritional intervention according to the dyslipidemia classification as proposed by Brazilian Society of Cardiology [32]. After applying the cut-off point for HDL-c, all groups showed similar changes. However, considering LDL-c subfractions, we observed a significant increase in LDL_LARGE_ in PsA patients from D + S group, but no impact on atherogenic LDL, a phenotype previously shown in individuals from general population after a low-carbohydrate diet ranging from 4 to 24 weeks [33, 34]. In another study [35], an intervention based on low-fat diet over 12-month period reduced the plasma LDL-c and increased HDL-c levels in healthy individuals. Di Minno et al. [36] demonstrated a reduction in hypercholesterolemia after a 6-month dietary intervention in PsA patients. However, there is some controversies regarding the effect of omega-3 fatty acids on LDL-c [37]. Gentile et al. [38] found higher concentration of small and dense LDL particles in 50 PsA patients than in control group, as well as a significant correlation between the LDL particles size and the carotid intima-media thickness, even in patients at low cardiovascular risk, suggesting a possible subclinical atherosclerosis. Recent studies have shown that small LDL particles are more easily oxidized and with greater affinity by extracellular matrix, lower receptor-binding, longer blood circulation and higher cardiovascular risk [39, 40].

It is worth emphasizing that almost 70% of our sample had an atherogenic pattern. However, if we consider the American Heart Association treat-to-target strategy according to the plasma LDL-c levels (< 130 mg/dL characterizing low or < 20% cardiovascular risk) [41], only 11 (11.2%) of our patients would be off-target. In addition, Ito et al. [42] reported an association between coronary artery disease and atherogenic LDL subfractions in individuals with plasma LDL-c levels below 100 mg/ dL, confirming that the lipoprotein profile may be more relevant than LDL-c itself. However, further prospective studies are needed to demonstrate whether these findings are associated with medium- and long-term cardiovascular outcomes, especially in patients with rheumatic diseases.

Our previous data suggest that PsA patients could have some benefits, such as quality lipoprotein subfractions and reduction of waist circumference and bodily adiposity measured by DXA after dietetic intervention and omega-3 fatty acids supplementation [19]. In fact, men who had higher BMI had more LDL_SMALL_ and atherogenic pattern, respectively, indicating that disease activity in PsA patients associated with obesity might maximize the inflammation itself [43].

Unexpectedly, the use of glucocorticoids in PsA patients under long-time treatment and older had lower atherogenic risk, differently from reported for patients with SLE and RA [44]. However, it is important to highlight that only 9 patients were on glucocorticoids to allow definite conclusions. Although pro-atherogenic pattern has been more common in patients with cardiovascular disease, the absolute risk of these events in long-term and the cut-off values of the LDL-c subfractions need to be addressed in further prospective studies [45]. On the other hand, Gentile et al. observed positive association between LDL_SMALL_ and disease activity and treatment [38]. Similarly, the LDL subfractions were influenced by disease activity, in which individuals with active synovitis had lower total cholesterol, LDL-, and HDL-cholesterol than controls [9].

Regarding the HDL subfractions, our results confirm the protective role of HDL_LARGE_ subfraction and higher cardiovascular risk for small particles, especially in men and in those with MetS [35]. According to our data, the protective HDL subfractions were more frequently found in patients with low disease activity, suggesting that the treat-to-target strategy for achieving remission could also improve the lipoprotein profile [46].

The total HDL-c and LDL-c plasmatic levels as well as their subfractions were similar between smokers and non-smokers, according to our results, unlike what was reported by Xi et al. [47]. Surprisingly, the statins did not significantly affect the lipoprotein profile after the nutritional intervention probably because these good effects should have been achieved earlier and no statin schedule changes were also allowed. Kucera et al. [35] demonstrated that statins reduced the LDL_SMALL_ particles and increased the LDL_LARGE_ particles in patients with hypercholesterolemia. These different results could have been found due to some aspects, such as lack of data concerning the adherence and treat-to-target to the statin therapy, a long time use without dosage modification and no very high values of cholesterol at baseline, suggesting that these patients were treated already. Additionally, it is worth noting that we were unable to match the current use of statins among the three groups during randomization, and this factor may have had an impact on the outcomes.

Interestingly, the DII from PsA patients was 2–3 times higher than observed in the adult healthy general Brazilian population [men: 1.12 (1.04); women: 1.24 (0.99)], suggesting that the rheumatologists should screen potential inflammatory foods and other relevant cardiovascular outcomes during the medical appointment and to refer to nutritionist to calculate it and to supervise suitable diet changes [48].

We adopted soybean oil as placebo because it represents the most frequent vegetable fatty acids font in the Western diet. Although the nutritional composition of soybean has some content of omega-6 and omega-3, our placebo group (1 g/d of soybean) added only 5% to the total habitual consumption of soybean oil in Western population (54.8 mL/day) [49]. In addition, soybean oil has steridonic acid (SDA), which is the precursor of EPA. However, the conversion rate in the body is limited, resulting in soybean not being a reliable source of EPA. The conversion from alpha-linolenic acid (ALA) to EPA is also inefficient, only approximately 5% of ALA is converted to EPA while less than 0.5% is converted to DHA in humans [50]. In comparison, each capsule of fish oil from our study contained 362 mg of EPA and 241 mg od DHA.

Another noteworthy point is that we did not exclude diabetic patients from this study since diabetes itself is a dyslipidemia risk factor. We decided to retain PsA patients with both conditions (MetS and diabetes were present in 54.6% and 20.6% in our sample, respectively) because they are frequently observed in clinical practice, enhancing external validity within a real-life setting. In addition, it is important to note that upon adjustments for MetS and diabetes, no significant differences were found among the three groups.

Our study has some limitations, such as the relatively short follow-up and small sample size. On the other hand, it has other strength points, including a modern methodology to evaluate the lipoprotein subfractions and a supervised and customized nutritional intervention clinical trial associated with omega-3 fatty acids supplementation in patients with PsA, as well as high compliance to the procedures and low dropout rate. Additionally, our findings were validated following adjustments for LDL- and HDL-cholesterol levelsby total and HDL-cholesterol, respectively. This approach aimed to mitigate potential biases introduced by non-paired total cholesterol plasmatic levels observed at baseline.

Finally, we concluded that lipoprotein subfractions assessment might improve the clinical screening and therapeutic monitoring in PsA patients over time, and nutritional intervention based on supervised and individualized health diet added to omega-3 fatty acids changed positively the HDL_LARGE_ subfractions, while LDL_LARGE_ subfraction was improved in hypercholesterolemic individuals. Therefore, we recommend adding lipoprotein subfractions analysis together to the early and traditional estimate of cardiovascular risk in PsA patients, as well as nutritional counseling and omega 3 supplementation in this scenario.

## Data Availability

The authors confirm that the data supporting the findings of this study are available within the article and its supplementary materials.
